# Foveal crack sign and macular pattern dystrophy

**DOI:** 10.1186/s40942-025-00655-8

**Published:** 2025-03-17

**Authors:** Rainer Rodriguez, David J. Browning

**Affiliations:** 1 Department of Ophthalmology, Wake Forest Bowman Gray School of Medicine, 475 Vine Street, Winston-Salem, NC USA; 2 Department of Ophthalmology, Atrium Health Wake Forest Baptist Medical Center, Winston-Salem, NC USA

**Keywords:** Optical coherence tomography, Macular hole, Hyperreflective foci, Retinal pigment epithelium, Vitelliform, Macula

## Abstract

**Purpose:**

To determine if eyes with the foveal crack sign (FCS) in macular pattern dystrophy (MPD) progress to macular holes, and if FCS occurs in the context of hyperreflective foci (HRF) that do not reach the threshold of FCS.

**Patients and methods:**

: A retrospective chart review of eyes with MPD was conducted in the Atrium Health Wake Forest Baptist medical system. 56 eyes from 32 patients identified as having macular pattern dystrophy were identified. Demographic data were collected from charts and spectral domain optical coherence tomographs (SD-OCTs) were analyzed.

**Results:**

8 eyes from 6 patients were found to have an FCS-positive OCT at any point at or following their initial diagnosis of MPD. One eye developed a full-thickness stage 4 macular hole during follow-up. There was no significant difference in macular hole development between MPD eyes with and without FCS (*p* =.1429). There was a significant difference in FCS development between MPD eyes with and without HRF (*p* =.0063).

**Conclusions:**

The data did not show a significant difference in macular hole generation between eyes with and without FCS, unlike the situation of FCS in eyes post vitrectomy for retinal detachment repair. The significant association between HRF and FCS suggests that the two signs may have a related pathophysiology and may represent different stages in a common degenerative process. Further work is needed to better characterize the relationship of FCS and HRF and to determine if FCS has different implications in the different clinical contexts in which it appears.

**Supplementary Information:**

The online version contains supplementary material available at 10.1186/s40942-025-00655-8.

## Introduction

Within the macula lies the fovea, the region of the retina with the highest concentration of cones and the greatest visual acuity [1]. The Foveal Crack Sign (FCS) is a recently described optical coherence tomography (OCT) finding that is characterized as a thin vertical and linear hyperreflectivity of the foveola from the internal limiting membrane to the ellipsoid zone [2, 3]. Other publications have described similar findings on OCT using different nomenclature including, but not limited to, hyperreflective stress lines (HSL), intraretinal hyperreflective lines (IHL) and hyperreflective foveal spots (HFS) [4–7].

Authors hypothesize pathophysiologic mechanisms for the sign largely involving two cell types – Muller cells and retinal pigment epithelium [2]. For Muller cells, traction is posited as altering orientation leading to hyperreflectivity; retinal pigment epithelial cells are thought to migrate inward as occurs in macular pattern dystrophies and other acquired vitelliform lesions, possibly causing pigment granule deposition and/or structural destabilization that appears hyperreflective on OCT [4, 6–10]. Ishibashi and colleagues [2] have emphasized that FCS reflects mechanical stress at the foveola; 77% of eyes in their series after vitrectomy surgery later developed full-thickness macular holes (FTMH). Other works have shown subsequent macular hole development in 50–80% of FCS-positive, non-post-surgical eyes [6, 11].

OCT biomarkers have been described as part of the natural course of eyes with vitelliform lesions, such as intraretinal hyperreflective foci (HRF) [10, 12]. Amoroso et al. [4] also described 49 eyes with macular linear hyperreflective lines; 24 had adult-onset foveomacular vitelliform dystrophy (AFVD) or other pattern dystrophy, and 17 of these linear hyperreflective lines were vertical. Multiple cases showcased in Amoroso et al. [4] satisfy the definition of FCS, and only macular microholes were reported, without statistical analysis.

**Fig. 1:**
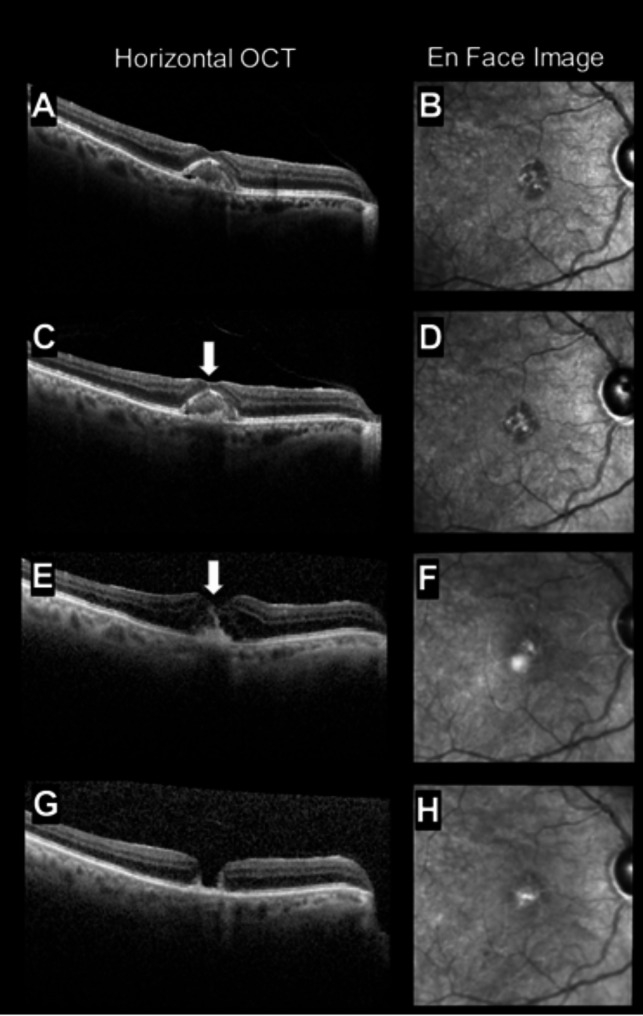
Case 7: (**A-B**) Eye presents with vitelliform lesion at first visit, as well as partial PVD that persists through multiple visits, (**C-D**) Foveal crack sign noted on second visit 4 months later, (**E-F**) Foveal crack sign remains present 9 months after initial visit, with absorption of vitelliform material, (**G-H**) full thickness macular hole on final visit, 7 months after presentation

Thus, little work has been done to further explore the relationship between hyperreflective OCT findings, MPDs, and subsequent development of macular holes. Determining the association between FCS, MPDs, and macular holes can clarify if FCS as a biomarker truly augurs macular holes or whether it is the post-vitrectomy state that is the proximate association with FCS as a fellow traveler, and when such patients should be followed closely by their ophthalmologist. Additionally, the relationship between FCS and other hyperreflective signs, such as HRF, has not been examined and may yield insights into the pathogenesis of FCS. Clinically, we have also noted FCS presence in patients with vitelliform dystrophies, represented in our current work by Case 7/Fig. 1. These unanswered questions, combined with clinical experiences, have inspired the present work.

In this study, we report on findings of patients diagnosed with macular pattern dystrophy whose OCT was found to have FCS. We also conducted a chart review of MPD patients to determine if FCS in these patients progressed to macular holes, if FCS occurs in the context of HRF, and any risk factors that may predispose patients to FCS development.

## Materials and methods

This retrospective chart review was conducted in the Atrium Wake Forest Baptist Health medical system and was conducted in accordance with IRB approval as IRB00113005. Research adhered to the tenets of the Declaration of Helsinki. Inclusion criteria were all patients who were diagnosed with a macular pattern dystrophy and had undergone retinal OCTs. “Macular pattern dystrophies” included AFVD, butterfly-shaped pattern dystrophy, reticular dystrophy, fundus pulverulentus, and multifocal pattern dystrophy simulating Stargardt disease. We confirmed diagnosis of macular pattern dystrophy utilizing chart review of available OCTs and any other retinal imaging, including but not limited to fluorescein angiography and color fundus photography. Exclusion of patients was made if they did not have an OCT available and a follow-up OCT of at least 6 months after the diagnosis of macular pattern dystrophy, or if other retinal diseases that could cause hyperreflective findings were present, such as age-related macular degeneration or diabetic retinopathy.

Charts were obtained by a search of the Wake Forest University Medical School and affiliated hospitals database. The database search was from the dates of 1/1/2000 to 5/1/2024, using the H35.50 ICD-10 code (ICD-10 code for MPD). All initial and follow-up ophthalmology visits were reviewed for inclusion and exclusion criteria. FCS was defined as a hyperreflective line extending from the ellipsoid zone towards the internal limiting membrane at the foveola, with case examples from Ishibashi et al. [2] and Furashova et al. [11] utilized as benchmarks for hyperreflectivity intensity and presentation. HRF were defined as hyperreflective dots or lesions that were not considered FCS; these were only noted within retinal layers of the foveal pit (maximum radius of 750 micrometers from the umbo). OCT images were reviewed for FCS and HRF; this was done by two reviewers independently, after which results were reviewed, and reviewers consolidated any differences. Macular holes were verified for presence and type by reviewing OCTs. All OCT’s were obtained as spectral domain OCTs (Heidelberg Spectralis), with distance between macular slices of 250 micrometers.

Data collected from charts included population descriptors such as age, sex, race, and eye laterality. Other data included past ocular history and intraocular surgical history, presence/type of MPD or macular hole, family/genetic history, best corrected visual acuity (BCVA) recorded as logMAR score, follow-up length in days from diagnosis to final visit, vitreous status and minimum macular thickness in microns. Data was analyzed initially using descriptive statistics. Comparison between groups was done using chi square tests for proportions and Fisher’s exact test. *P* values less than 0.05 were judged to be statistically significant.

## Results

From the database search, 250 eyes were initially identified as possible MPD cases. Following verification of inclusion and exclusion criteria, 56 eyes from 32 patients were found to have MPD. Of these, 8 eyes from 6 patients were found to have an FCS-positive OCT at any point at or following their initial diagnosis of MPD. Descriptive characteristics for these are shown in Table 1. Mean patient age was 70 ± 10 years old, and the mean length of follow-up was 1106 ± 492 days. There was no genetic testing available for all cases.

**Table 1:** OD = Right; os = left; AFVD = Adult-onset foveomacular vitelliform dystrophy; a – Patient-reported where 1 = closest affected relative within nuclear family and 2 = closest affected relative within extended family; b – patient had a Pars plana vitrectomy for retinal detachment during follow-up (299 days after foveal crack sign presentation)

Case	Sex/Age	Race	Eye Laterality	Macular Pattern Dystrophy Subtype	Family History?^a^	Intraocular Surgical History?	Length of follow-up (in days)
1	F/68	White	OD	AFVD	1	No	1372
2	F/80	White	OD	AFVD	0	No	1495
3	F/80	White	OS	AFVD	0	No	1495
4	F/73	White	OD	AFVD	1	No	1324
5	F/73	White	OS	AFVD	1	No	1324
6	F/56	White	OD	Unspecified	1	No^b^	325
7	F/79	White	OD	AFVD	2	No	324
8	M/54	White	OD	Unspecified	0	No	1187

Table 2 Shows further collected measures in FCS-positive eyes. BCVA had a mean change of 0.295 ± 0.588 from initial visit to final visit. Macular thickness had a mean change in thickness of -33.6 ± 61.9 microns from initial to final visit. The mean time from initial visit to FCS presentation was 373 ± 351 days. One eye developed a full-thickness stage 4 macular hole during the follow-up period.

**Table 2:** Best corrected visual acuity (BCVA) in LogMAR; Foveal Thickness in micrometers; a – PVD = posterior vitreous detachment; b – noted in fundal exam but not seen on review of OCT; c - Regressed into foveal hyperreflective foci; d - dislocated intraocular lens; e - macular hole; f - Developed into macular hole; g - thickness measured using asymmetry analysis rather than thickness map change report on Heidelberg

Case	Initial BCVA	Final BCVA	Initial Minimum Central Foveal Thickness	Final Minimum Central Foveal Thickness	Days from Initial Visit to FCS	Did FCS resolve, change, or remain stable?	Vitreous Status on FCS arrival	Presence of Epiretinal Membrane at FCS diagnosis	Macular Hole during Follow-Up?/Days from FCS
1	0.4	0.06	255	240	722	Resolved	PVD^ab^	None	N
2	0.48	0.52	340	327	645	Changed^c^	PVD^b^	Present	N
3	0.6	0.42	348	343	988	Stable	PVD^b^	Present	N
4	0.7	1.8^d^	364	225	162	Stable	Attached	None	N
5	1	1	221	207	162	Resolved	Attached	None	N
6	0.24	0.4	298	272	36	Changed^c^	PVD	None	N
7	0.52	1.8^e^	334	223	135	Changed^f^	Partial PVD	None	Y/176
8	1	1.3	225	279^g^	137	Stable	Attached	None	N

**Fig. 2:**
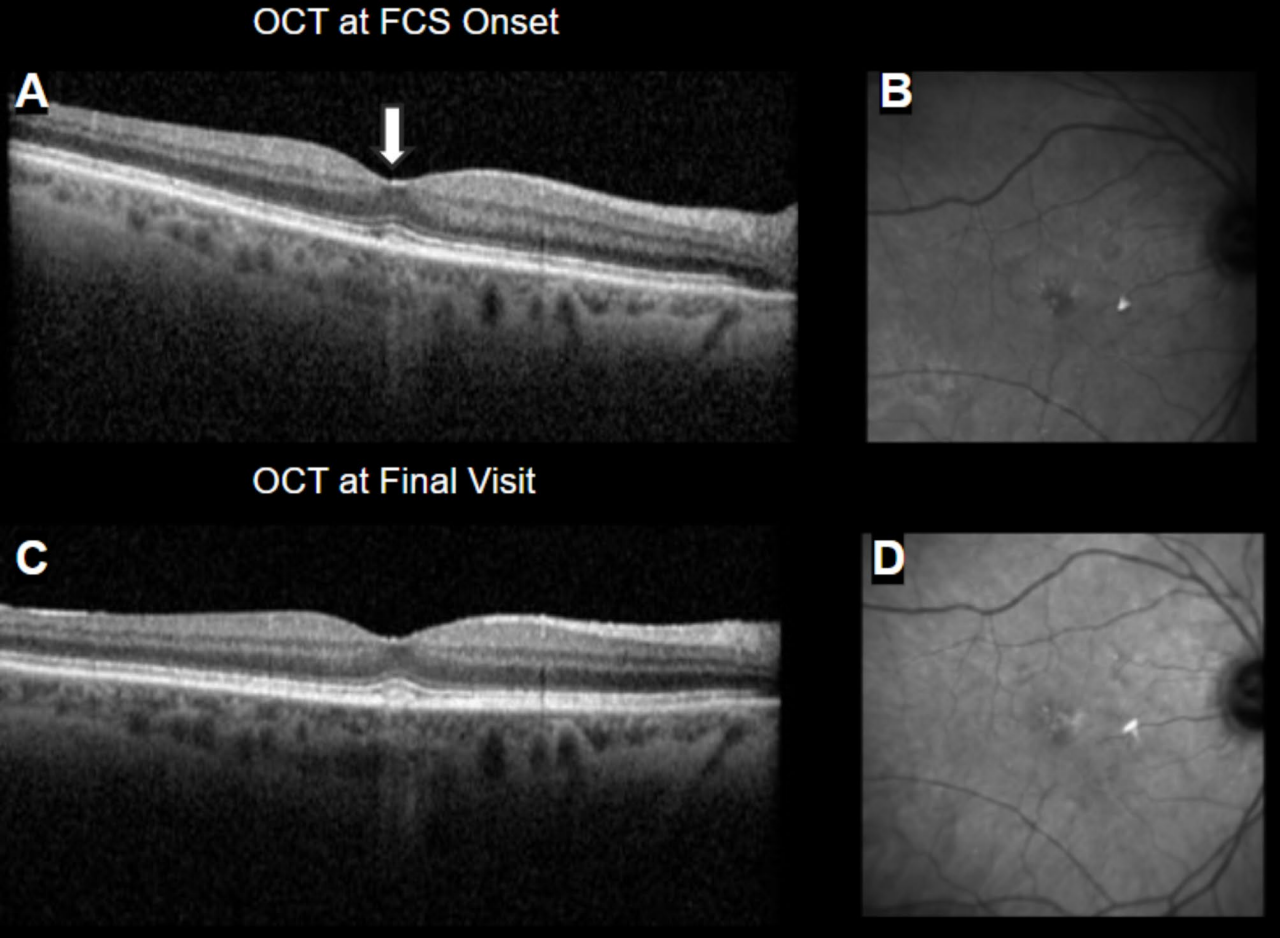
Case 1: (**A-B**) Mild foveal crack sign marked by white arrow, (**C-D**) disappeared by final visit

**Fig. 3:**
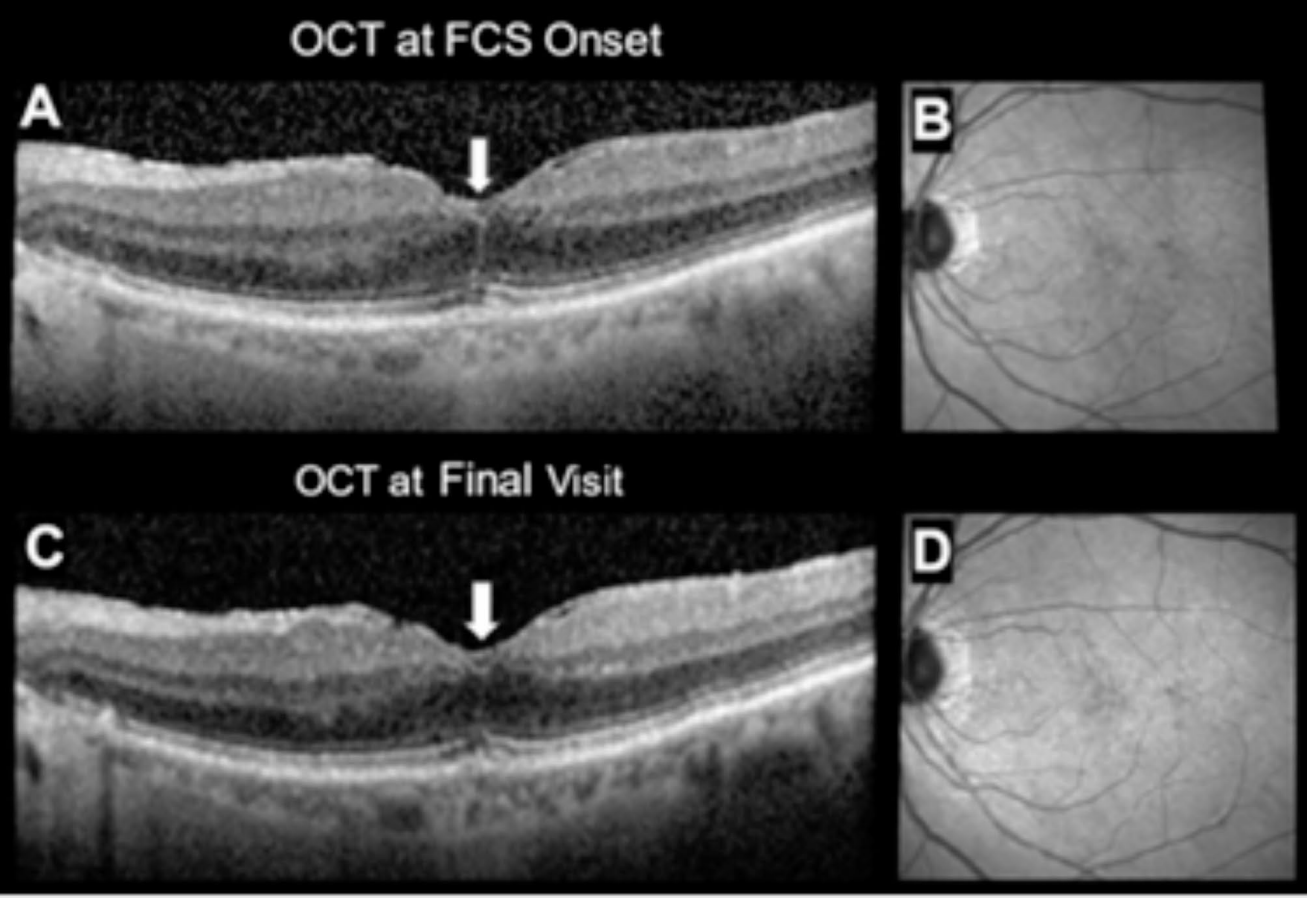
Case 3: (**A-B**) Moderate foveal crack sign denoted by white arrow, (**C-D**) mild foveal crack sign still present by final visit

**Fig. 4:**
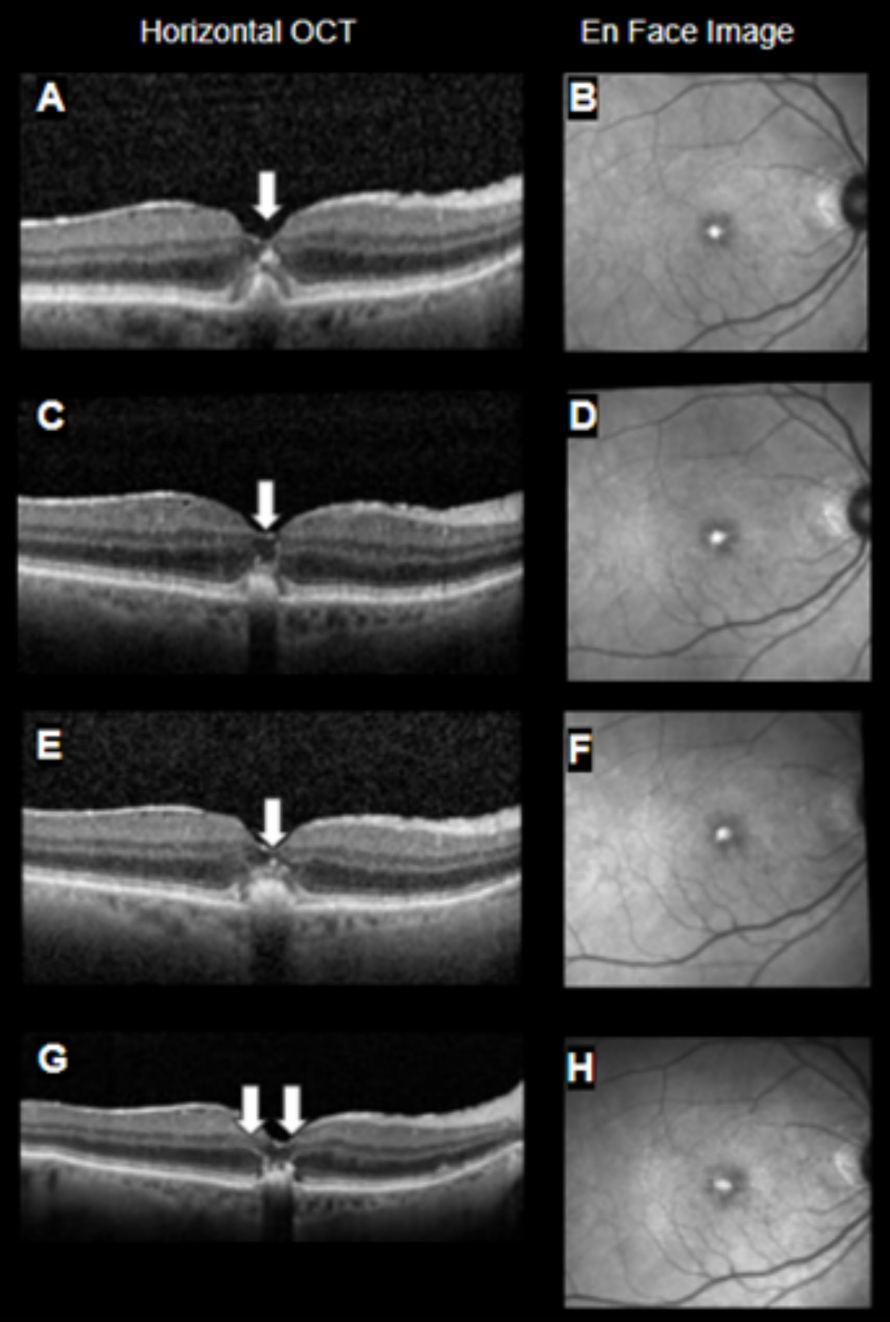
Case 2: (**A-B**) Patient seen at the one year follow-up visit with foveal hyperreflective foci denoted by white arrow, (**C-D**) Foveal crack sign noted 10 months later and denoted by arrows, alongside a small hyperreflective foci, (**E-F**) loss of foveal crack sign one year after identification, multiple foveal hyperreflective foci denoted by white arrow, (**G-H**) Angular sign of Henle fiber layer hyperreflectivity denoted by arrows, without foveal crack sign 4 years after presentation

Figures 1, 2, 3, and 4 display OCT’s from 4 representative eyes. Fig. 1 displays the development of FCS that progressed to a full-thickness macular hole. Figure 2 shows an eye that presented with and lost the FCS by the end of follow-up. Figure 3 shows an eye that developed and maintained the FCS by end of follow-up. Finally, Figure 4 shows the development of hyperreflective congeners, as well as the development and resolution of FCS.

There was no difference in rate of macular hole development between MPD eyes that had or did not have a FCS (*P* =.143, Table 3). There was an association between HRF and FCS (*P* =.0063, Table 4).

**Table 3:** Contingency table for Foveal Crack Sign and Macular Holes

	Development of Macular Hole	No Development of Macular Hole
Presence of FCS	1	7
No presence of FCS	0	48

**Table 4:** Contingency table for Foveal Crack Sign and Hyperreflective Foci

	HRF Present	HRF Absent
Presence of FCS	8	0
No presence of FCS	23	25

## Discussion

Only one of the eyes reviewed developed a macular hole following identification of FCS on OCT. Table 3 showed that there was no significant difference in macular hole development between MPD eyes with and without FCS. Thus, in MPD patients, finding FCS on routine OCT might not augur macular hole development as has been noted for patients with FCS after vitrectomy to repair retinal detachment [2]. The data showed an association between the presence of FCS and the presence of HRF, suggesting that the two signs may be related pathophysiologically.

Our results run contrary to the literature on FCS. Ishibashi et al. [2] looked at post-surgical pars plana vitrectomy eyes, compared to our sample of MPD eyes. Furashova et al. [11] studied fellow eyes of eyes with macular holes and found FCS in 10/19 fellow eyes of FTMH. Scharf et al. [6] looked at eyes that developed FTMH and lamellar macular holes and found FCS preceded FTMH in 50% and lamellar macular holes in 25%, respectively. Kayabasi et al. [7] characterized the presence of IHLs in multiple pathologies and found that, of 40 eyes, 35% had undergone vitreoretinal surgeries prior to IHL formation, mostly for FTMH. The absence of a history of vitrectomy in the eyes in our series may explain the differences in rates of subsequent macular hole compared to the series of Ishibashi et al. [2] and Scharf et al. [6]. Also, the use of fellow eyes in Furashova et al. [11] may have made their sample more likely at baseline to develop a macular hole than the eyes analyzed in the present study.

The main clinical implication from this study is that FCS could have a low positive predictive power for macular hole progression in non-vitrectomized eyes, such as MPD, and that more work is needed to both further characterize FCS and analyze its value in clinical decision making. FCS may be an imaging finding that is sensitive, but not specific, to macular hole development. It is also worth noting that in MPD patients, disruption of the normal foveal contour can increase interpretational difficulty in reading OCTs.

Our findings could support the hypothesis of FCS as a possible subtype of HRF by Iwama et al. [9] However, more work is needed as we cannot rule out that FCS and HRF are independent processes confounded by similar risk factors and conditions. It is worth noting that congeners such as foveal HRF’s are more common in pathologic eyes and can also mimic FCS, increasing the difficulty of characterizing a true FCS and possibly limiting FCS utility.

Multiple limitations to the data are worth discussion. This study arose from questions the research team had regarding FCS-positive eyes with MPD seen in the clinical setting, and whether these eyes were at greater risk of macular hole formation following FCS identification. This was a possible association that had not yet been explored in the literature, despite evidence that FCS could occur in MPDs and augur macular hole formation [2, 4, 11]. We set out to answer these specific questions, accepting the limitations of MPD as a less prevalent diagnosis, relative to other retinal pathologies such as age-related macular degeneration and diabetic retinopathy. Our work should be interpreted in this context, as this limits both the generalizability and power of our analysis. For instance, almost all our FCS-positive eyes were diagnosed as cases of AFVD, raising questions on if other forms of MPD may display a different natural history following FCS identification. With regards to sample size, the use of MPD, in concert with an infrequent biomarker like FCS, limits the number of cases we were able to identify. Additionally, our FCS-positive eyes consisted of only Caucasians and might not be an accurate representation of the utility of FCS in other races with MPD. We encourage further work analyzing the relationship between FCS and hole formation in more prevalent and homogenous retinal pathologies, which may increase power and generalizability of results.

There are also limitations in identifying FCS itself, as it can be subtle [11]. Artifacts on OCT scans can mimic or obscure FCS, making the interpretation of the OCT in MPD patients more difficult than in unaltered maculas. In addition, there are FCS mimickers such as parafoveal crack signs and the angular sign of Henle fiber layer hyperreflectivity (ASSH) [13]. These mimickers present at obtuse angles to the umbo, and their utility or hindrance to prognostication of future macular health is unclear. We also noted artifacts that mimicked FCS arising from nonorthogonal positioning of the macula relative to the scan beam OCT may vary across patients and imagers; nonstandardized brightness settings and aspect ratios invite image variation than can resemble FCS.

Questions remain about the pathogenesis of FCS and other hyperreflective OCT biomarkers in MPD, especially when tractional forces such as vitreomacular traction and epiretinal membrane are not present. The hypotheses extant regarding the pathophysiology of the foveal crack sign are as follows:

1. Muller cell damage from traction, ischemia, or metabolic dysfunction in the underlying retinal pigment epithelium leading to localized structural instability [2, 4, 8, 11].
2. Outer retinal and retinal pigment epithelial misalignment with disruption of the outer retina and retinal pigment epithelium interface leading to the sign [2, 4, 11].
3. Chronic macular edema with an atrophic or fibrotic remodeling response [14].
4. Manifestation of early lamellar macular holes and foveal thinning [6, 11].

Ucar et al. [15] found a loss of parafoveal vascular density in both butterfly-pattern dystrophy and adult foveomacular vitelliform dystrophy on OCTA compared to controls; there was no difference in the areas of the foveal avascular zones [14]. We also know that structural and retinal pigment epithelial integrity are impaired in pattern dystrophies, seen as geographic macular atrophy, decreased RPE height and packing density and decreased photoreceptor packing density on multimodal imaging [16, 17]. Chen et al. [10] have found that HRF associated with acquired vitelliform lesions are represented on histopathology by migrated RPE cells and pigment granules. We posit that Mechanisms 1, 2, and 4 are potentially relevant in FCS associated with MPD. Mechanism 3 is not germane, as there is no chronic edema associated with MPD.

We posit that decreased parafoveal vascular density may disrupt foveal homeostasis and structural integrity, creating a susceptibility to future tractional forces that, in combination with retinal pigment migration and granule deposition, results in a propensity for vertical linear foveolar hyperreflectivity on OCT, seen as FCS, in eyes with acquired vitelliform lesions. This multimodal theory of pathogenesis may be supported by the presence of ERM in 38% of our FCS-positive cases, as well as multiple stages of vitreous attachment. In our study, Case 7 developed FTMH and notably had a partial PVD with a large vitelliform lesion that was enhanced following vitelliform lesion absorption. Regression of FCS may represent a loss of tractional forces combined with the completed resorption of migrated material. These hypotheses require further study.

There are follow-up questions that arise from our findings. Studies with larger sample sizes should be conducted to better analyze the sensitivity and specificity of FCS with regards to full-thickness and especially lamellar macular holes, as well as other macular pathology such as maculoschisis and cystoid macular edema. The prevalence of the FCS in retinitis pigmentosa, Best disease, Stargardt disease, diabetic retinopathy and age-related macular degeneration should be evaluated. Determining whether the FCS implies a different outcome in post-surgical patients should also be further investigated. The relationship of hyperreflective foci, partial FCS, congeners such as ASSH, and full FCS needs further exploration as well.

## Conclusion

FCS in MPD did not predict subsequent macular hole formation. Further work is needed to better characterize FCS and its congeners and to determine if it has different implications in different clinical contexts.

## Electronic supplementary material

Below is the link to the electronic supplementary material.

Supplementary Material 1

## Data Availability

Raw, deidentified data is provided in the supplementary information files.

## References

[1] Levin L, Kaufman P, Hartnett ME. Adler’s physiology of the eye. 12th ed. Elsevier; 2024.

[2] Ishibashi T, Iwama Y, Nakashima H, Ikeda T, Emi K. Foveal crack sign: an OCT sign preceding macular hole after vitrectomy for rhegmatogenous retinal detachment. Am J Ophthalmol. 2020;218:192–8. 10.1016/j.ajo.2020.05.030. 32479809 10.1016/j.ajo.2020.05.030

[3] Pandya BU, Grinton M, Mandelcorn ED, Felfeli T. RETINAL OPTICAL COHERENCE TOMOGRAPHY IMAGING BIOMARKERS. Retina. 2024;44(3):369–80. 10.1097/IAE.0000000000003974. 37903455 10.1097/IAE.0000000000003974 PMC10885864

[4] Amoroso F, Mrejen S, Pedinielli A, et al. INTRARETINAL HYPERREFLECTIVE LINES. Retina. 2021;41(1):82–92. 10.1097/IAE.0000000000002806. 32251237 10.1097/IAE.0000000000002806

[5] Pierro L, Arrigo A, Capone L, et al. Hyperreflective foveal spots in patients with vitreoretinal anomalies. Retina. 2020;40(4):705–9. 10.1097/IAE.0000000000002505. 30845025 10.1097/IAE.0000000000002505

[6] Scharf JM, Hilely A, Preti RC, et al. Hyperreflective stress lines and macular holes. Invest Opthalmology Visual Sci. 2020;61(4):50. 10.1167/iovs.61.4.50. 10.1167/iovs.61.4.50 PMC7401923 32347919

[7] Kayabasi M, Koksaldi S, Saatci AO. Intraretinal hyperreflective line: potential biomarker in various retinal disorders. Med Hypothesis Discovery Innov Ophthalmol. 2024;13(3):129–38. 10.51329/mehdiophthal1504. 10.51329/mehdiophthal1504 PMC11537236 39507809

[8] Arrigo A, Marchese A, Pierro L, Bandello F. Comment on: foveal crack sign: an optical coherence tomography sign preceding macular hole after vitrectomy for rhegmatogenous retinal detachment. Am J Ophthalmol. 2020;219:366. 10.1016/j.ajo.2020.06.037. 32948294 10.1016/j.ajo.2020.06.037

[9] Iwama Y, Ishibashi T, Nakashima H, Ikeda T, Emi K. Reply to comment on: foveal crack sign: an optical coherence tomography sign preceding macular hole after vitrectomy for rhegmatogenous retinal detachment. Am J Ophthalmol. 2020;219:367. 10.1016/j.ajo.2020.06.036. 32917372 10.1016/j.ajo.2020.06.036

[10] Chen KC, Jung JJ, Curcio CA, Balaratnasingam C, Gallego-Pinazo R, Dolz-Marco R, Freund KB, Yannuzzi LA. Intraretinal hyperreflective foci in acquired vitelliform lesions of the macula: clinical and histologic study. Am J Ophthalmol. 2016;164:89–98. 10.1016/j.ajo.2016.02.002. 26868959 10.1016/j.ajo.2016.02.002

[11] Furashova O, Matthé E. Foveal crack sign as a predictive biomarker for development of macular hole in fellow eyes of patients with full-thickness macular holes. Sci Rep. 2020;10(1):19932. 10.1038/s41598-020-77078-y. 33199791 10.1038/s41598-020-77078-y PMC7670431

[12] Fragiotta S, Abdolrahimzadeh S, Dolz-Marco R, Sakurada Y, Gal-Or O, Scuderi G. Significance of Hyperreflective Foci as an Optical Coherence Tomography Biomarker in Retinal Diseases: Characterization and Clinical Implications. Milani P, ed. J Ophthalmol. 2021;2021:1–10. 10.1155/2021/6096017 10.1155/2021/6096017 PMC8709761 34956669

[13] Ramtohul P, Cabral D, Sadda S, Freund KB, Sarraf D. The OCT angular sign of Henle fiber layer (HFL) hyperreflectivity (ASHH) and the pathoanatomy of the HFL in macular disease. Prog Retin Eye Res. 2023;95:101135. 10.1016/j.preteyeres.2022.101135. 36333227 10.1016/j.preteyeres.2022.101135

[14] Podkowinski D, Philip A, Vogl W, et al. Neuroretinal atrophy following resolution of macular oedema in retinal vein occlusion. Br J Ophthalmol. 2019;103:36–42. 29511062 10.1136/bjophthalmol-2017-311614

[15] Uçar D, Kılıçarslan O, Yılmaz Çebi A. Quantitative microvascular alterations in butterfly-shaped pattern dystrophy and adult-onset foveomacular vitelliform dystrophy. J Français d’Ophtalmologie. 2024;47(2):103977. 10.1016/j.jfo.2023.08.009. 10.1016/j.jfo.2023.08.009 37845141

[16] Marmor MF, Mcnamara JA. Pattern dystrophy of the retinal pigment epithelium and geographic atrophy of the macula. Am J Ophthalmol. 1996;122(3):382–92. 10.1016/S0002-9394(14)72065-3. 8794711 10.1016/s0002-9394(14)72065-3

[17] Liu T, Aguilera N, Bower AJ, Li J, Ullah E, Dubra A, Cukras C, Brooks BP, Jeffrey BG, Hufnagel RB, Huryn LA, Zein WM, Tam J. Photoreceptor and retinal pigment epithelium relationships in eyes with vitelliform macular dystrophy revealed by multimodal adaptive optics imaging. Invest Opthalmology Visual Sci. 2022;63(8):27. 10.1167/iovs.63.8.27. 10.1167/iovs.63.8.27 PMC9344216 35900727

